# Current concepts regarding developmental mechanisms in diabetic retinopathy in Taiwan

**DOI:** 10.7603/s40681-016-0007-3

**Published:** 2016-05-05

**Authors:** Shih-Yin Chen, Yuan-Man Hsu, Ying-Ju Lin, Yu-Chuen Huang, Chao-Jung Chen, Wei-De Lin, Wen-Lin Liao, Yng-Tay Chen, Wei-Yong Lin, Yu-Huei Liu, Jai-Sing Yang, Jinn-Chyuan Sheu, Fuu-Jen Tsai

**Affiliations:** 1 Genetics Center, Department of Medical Research, China Medical University Hospital, No. 2 Yuh Der Road, 404 Taichung, Taiwan; 2 School of Chinese Medicine, China Medical University, 404 Taichung, Taiwan; 3 Department of Biological Science and Technology, China Medical University, 404 Taichung, Taiwan; 4 Institute of Biomedical Sciences, National Sun Yat-sen University, 804 Kaohsiung, Taiwan; 5 Department of Medical Genetics, China Medical University Hospital, 404 Taichung, Taiwan

**Keywords:** Diabetic retinopathy (DR), Retina, Inflammation, Oxidative stress, Vascular damage

## Abstract

Diabetic retinopathy (DR) is one of the most feared complications of diabetes and is a leading cause of acquired blindness in working adults. The prevalence of undiagnosed diabetes in Taiwan is about 4%, and the annual incidence of T2D (Type 2 Diabetes) in Taiwan is 1.8% following the 1985 WHO criteria. Multiple mechanisms have been shown in T2DR with some signaling pathways, including the polyol pathway, PKC pathway, AGEs pathway, and MAPK pathway. However, the cause of vision loss in diabetic retinopathy is complex and remains incompletely understood. Herein, we try to fully understand the new concepts regarding hyperglycemia-induced biochemical pathways contributing to DR pathophysiology. Our work may be able to provide new strategies for the prevention and treatment of diabetic vascular complications.

## 1. Introduction

Diabetes mellitus (DM) is a complex disease that is caused by absolute or relative insulin deficiency. According to the World Health Organization (WHO), in 2014, around 350 million people had DM, and it will be the seventh leading cause of death in 2030 [1]. India, China, and the US are projected to have the largest number of people with DM by 2025. Following the 1996 WHO criteria, the prevalence of undiagnosed diabetes in Taiwan is about 9%, and the annual incidence of type 2 DM (T2D) in Taiwan is 1.8% [2]. In Taiwan, there are about 540 thousand drug-treated diabetes patients who combined cost about 11.5% of the total health care expenditure that is reimbursed through Taiwan’s National Health Insurance. According to epidemiological surveys, the prevalence of diabetes increased gradually from 5.05% to 7.10% to 8.17% in 1970, 1979, and 1986, respectively, for those over 40 years of age [3-6]. In more recent years, the prevalence of diabetes has been found to be from 6.5% to 12.4% in community- based studies in Taiwan for the resident population aged 30 years or older [7, 8]. Adults demonstrated an increasing trend in T2D during 1999-2004 in Taiwan [9]. T2D has become one of the major health problems in Taiwan. This disease is mainly divided into 4 groups: type 1 DM (T1D), T2D, and other specific types of DM and gestational DM (GDM). None of these 4 types of DM is a single homogeneous disease. T2D accounts for 90% of all diabetic patients at present [1]. Previous studies have identified different risk factors for increasing the incidence of DM like age, body mass index (BMI), systolic blood pressure (BP), fasting plasma glucose, glycated hemoglobin (HbA1c), *etc*. Clinically, DM expresses hyperglycemia, dyslipidemia, hyperhomocysteinemia, and decreased β-cell or insulin function and section to induce insulin resistance [10]. Long-term metabolic dysfunctions are caused by various complications such as microvascular disease, diabetic neuropathy, diabetic retinopathy (T2DR), and dementia [11-13]. Retinopathy is rarely detected in the first few years of DM, while by 10 years it is approximately 50% of the DM, and near 90% of DM by 20-25 years [9]. When visual problems start to appear, retinopathy has advanced to a point where it cannot be treated and cured. T2DR is the most common cause of acquired blindness in diabetic adults. Lifestyle modifications and nutritional adjustments are some of the best methods for T2D prevention and treatment. Genetic factors have also been shown as new ways to fight against T2D and play an important role in DM [14]. This review integrates several candidate genes and pathways whose implications have been previously discovered and discussed in patients with diabetic retinopathy in Taiwan.

## 2. Pathogenesis of DR

In using fasting plasma glucose (FPG) levels as a means of diagnosis, the age-adjusted incidence rate of T2D was 0.89% per year according to research done on the 35-74 years of age cohort that was conducted once from 1990 to 1993 and again from 1993 to 1996 in Taiwan [15]. Hyperglycemia induced complex metabolic abnormalities, such as systemic abnormalities (retinal bloods’ alteration, homeostatic abnormalities), oxidant stress, and inflammation pathway activation. Increased vascular permeability, hemostatic abnormalities, endothelial dysfunction, increased tissue ischemia, and neoangiogenesis are all characteristics of T2DR [16]. T2DR can be classified into 2 stages: (1) nonproliferative T2DR, which involves the leakage of retinal vessels into surrounding tissue, the thickening of the basement membranes, as well as the induction of microaneurysms, retinal hemorrhage, and capillary nonperfusion; and (2) proliferative T2DR, which is when retinal cells start to undergo accelerated inflammation, expansion of extracellular matrix (ECM), the surface of the retina grows new blood vessels, and apoptosis occurs. These abnormal vessels easily bleed and then vitreous hemorrhaging, fibrosis, and retinal detachment occurs [17]. Therefore, the clinical feature of HbA1c in blood is the most important biochemistry related to the prevalence of T2DR in Taiwanese T2D patients [18]. Stableblood pressure and glycemic are also modifiable factors in the prevention of T2DR. Both environmental (glycemic control, BP, duration of diabetes, *etc*.) and genetic factors are important to the development of T2DR. The molecular mechanisms of T2DR along five major signaling pathways have been comprehensively discussed. They are (1) the polyol pathway (sorbitol-aldose reductase pathway), (2) the protein kinase C (PKC) pathway, (3) the advanced glycation end products (AGEs) pathway, (4) the hexosamine pathway, and (5) the mitogen-activated protein kinase (MAPK) pathway. The high glucose-related damage affects specific tissues (such as retina, kidney, and nerve tissues) where insulin is not required for cellular glucose uptake during diabetes because cells in these tissues are deficient in their ability to change how they transport glucose and thus lose their glucose balance[13, 19]. Glucose is primarily metabolized and oxidized by tricarboxylic acid (*via* the TCA cycle) and this produces nicotinamide adenine dinucleotide (NADH) and Flavin adenine dinucleotide (FADH_2_) which both flux into the mitochondrial electron transport chain and adenosine triphosphate (ATP) generation that accompany superoxide production. The mitochondria-reactive oxygen species (ROS) pathway through increase H_2_O_2_ generation and reduce UCP2 (uncoupling protein-2) expression, associated with decrease cytochrome oxidase, Mn-SOD (mitochondrial superoxide scavenging enzyme) and NOS activities, leading to impaired membrane mitochondrial potential [20, 21].

### 2.1. Polyol pathway

**Figure 1:**
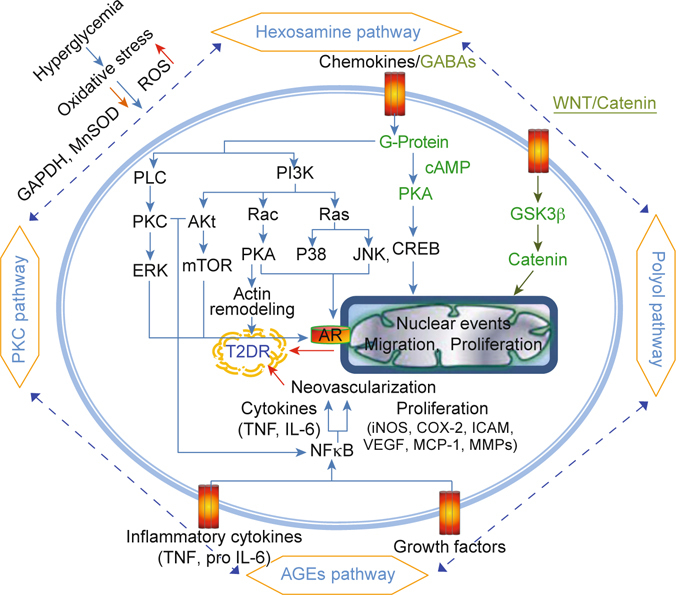

The polyol pathway is a two-step metabolic pathway: glucose is first reduced to sorbitol and then converted to fructose when intracellular glucose levels are elevated [21]. The rate limiting enzyme in the polyol pathway is aldose reductase (AR), which reduces unused glucose to sorbitol, then sorbitol dehydrogenase (SDH) oxidizes sorbitol to fructose, and then nicotinamide adenine dinucleotide phosphate (NADPH) oxidizes to nicotinamide adenine dinucleotide phosphate (NADP^+^). Sorbitol almost never diffuses through cell membranes and this results in its accumulation, causing osmotic damage [16]. The NADPH/NAD^+^ ratio is reduced through reduction-oxidation reactions of reduced glutathione (GSH) to oxidized glutathione (GSSG). NADPH deficiency inhibits the production of nitric oxide (NO), increases ROS accumulation, and stimulates diacylglycerol (DAG) synthesis. Hyperglycemia induces tonicity-responsive enhancer binding protein (TonEBP, a transcription factor) levels to increase AR and protein kinase C δ (PKC δ) levels, which was shown to lead to apoptotic death in a mouse model of diabetic retinopathy [22] (Figure 1). In the study, an extract of purple waxy corn and ginger combined prevented cataractogenesis and retinopathy in streptozotocin-diabetic rats by decreasing lens opacity, malondialdehyde (MDA), and AR in the lens and by enhancing catalase (CAT), and glutathione peroxidase (GPx) activities, thereby increasing the number of neurons in the ganglion cell layer and the thickness of the total retina and the retinal nuclear layer [23].

### 2.2. PKC pathway

Various PKC isoforms have been reported to be changed in vascular cells by diabetes or an otherwise increased glucose level. PKCα, PKCβI, PKCβII and PKCε isoforms have been reported to be enhanced in the retina membrane of diabetic rats. PKCβ1/2, meanwhile, exhibited a significant increase in the membrane fraction of all vascular tissues. When exposed to elevated glucose levels, PKCβII and PKCδ have been shown to be improved in bovine capillary retinal endothelial cells. Phosphorylation of PKCα and PKCβI have been found to prevent glomerular dysfunction in diabetic rats [24]. PKC activation takes part in multiple diabetic complications including changes in blood flow, basement membrane thickening, extracellular matrix expansion, vascular permeability, angiogenesis, cell growth, and enzymatic activity alteration (MAPK). DAG is an important second messenger and activates PKC. Some reports have suggested that PKC-selective inhibitors cause a decrease in PKC activity and DAG levels that may improve motor nerve conduction velocity and endoneurial blood flow in diabetic animals [25, 26]. PKC activation decreases nitric oxide (NO) production *via* endothelial nitric oxide synthase (eNOS) activity in blood flow and leads to glomerular hyperfiltration. In contrast, PKC activity up-regulates the expression of the transforming growth factor β (TGF-β) and the nuclear factor kappa- light-chain-enhancer of activated B cells (NF-κB). This causes ECM proteins to remodel and then increases levels of basement membranes. PKC activation induces vascular endothelial growth factor (VEGF), leading to macular edema and proliferative retinopathy [21]. Angiogenic factors increase and release endothelial and leukocyte dysfunction, which may lead to capillary occlusion, as well as changes in blood flow to the retina [27] (Figure 1).

### 2.3. AGEs (advanced glycation end products) pathway

The causal relationship between chronic inflammation and angiogenesis in T2DR is widely accepted. AGEs play a role in this relationship as proinflammatory mediators in retinopathy in which chronic exposure of the retina in hyperglycemia is increased. AGEs are proteins or lipids that come from the glycation reaction, which refers to the addition of a carbohydrate to a protein with nonenzymatic reaction. AGE formation (carboxyethlpyrrole and MDA) with higher expressions of AGE receptors (galectin-3, and CD-36) is also evidenced in the retinal vessels of patients with T2DR [13]. The binding of AGE receptors (RAGE) can start important signaling pathways involving tyrosine phosphorylation of Janus kinase (JAK)/signal transducers, activators of transcription (STAT), recruitment of phosphatidylinositol 3 kinase to Ras, activation of PKC, and oxidative stress through NFκB and activator protein-1(AP-1) transcription [28]. Hyperglycemia induces hypoxia in retinal tissue, which attracts growth factor (VEGF), erythropoietin (EPO), adhesion molecules [intercellular adhesion molecule (ICAM-1), vascular cell adhesion molecule (VCAM-1)], cytokines [vascular adhesion protein (VAP-1)], and inflammatory genes [tumor necrosis factor-α (TNF-α), interleukin-1β (IL- 1β), and interleukin-8 (IL-8)]. The signal transductions will be regulated by those genes which were mentioned in this section and lead to angiogenesisin in T2DR development. VEGF is one of the major mediators in the intraocular neovascularization of T2DR [21] (Figure 1).

### 2.4. Hexosamine pathway

*In vitro* and *in vivo* studies have revealed insulin resistance and diabetic vascular complications *via* the hexosamine pathway to the flux of glucose. In the hexosamine pathway, fructose 6-phosphate (F-6-P) is converted to Glucosamine 6-phosphate by fructose 6-phosphate amidotransferase (GFAT, rate-limiting enzyme). Glucosamine 6-phosphate is afterward converted to uridine diphodphate-N-acetylglucosamine (UDP-GlcNAc; attaches to transcription factors). UDP-GlcNAc is a high-energy molecule that serves as the monosaccharide donor for the posttranslational modification of the substrate for O-linked GlcNAc (O-GlcNAc) by O-GlcNA transferase (OGT). O-GlcNAcase (OGA) removes the O-GlcNAc modification of proteins. O-GlcNAc protein modifications change the implication of postnatal retinal vascular development and the pathogenesis of T2DR patients [29]. T2DR inhibits GFAT blocks and increases the transcription factors [transcription growth factor (TGF-α), TGF-β1, and plasminogen activator inhibitor-1 (PAI-1)], leading to posttranslational modification [13] (Figure 1).

### 2.5. Matrix metalloproteinases (MMPs) pathway

Matrix metalloproteinases (MMPs) are large zinc-dependent endogenous proteolytic enzymes that remodel extracellular matrix components. MMPs appear to play multiple roles in the regulation of a variety of cellular functions, including apoptosis and angiogenesis, and inflammation [30]. There is evidence of higher lipid peroxidation, superoxide production, and lower activity of antioxidant enzymes in the retina during the development of T2D. One of the important molecular mechanisms of oxidative stress is the regulation of MMPs. T2D is believed to stimulate the secretion of several MMPs, which participate in both macro- and microvascular abnormality-associated diseases (like retinopathy) [31]. MMPs may be activated by ROS in patients and animal models with diabetic retinopathy, which have shown increased MMP-9 and MMP-2 in their retina and vitreous [32, 33]. MMPs are supposed to be a negative regulator of cell survival. MMP-2 and -9 expressions might be made *via* the Ras/Raf ERK1/2 and PI3K/ Akt/mTOR pathways to induce diabetic retinopathy. Activated Raf subsequently phosphorylates MAPK kinase 1/2 (MEK 1/2), which then phosphorylates two substrates, extracellular signal regulated kinase 1/2 (ERK1/2) [34, 35]. On the other hand, Activated PI3K subsequently phosphorylates AKT, which then phosphorylates two substrates, extracellular signal regulated mTOR complex [36]. Latent activation of MMP-2 and MMP-9 induce retinal changes and the apoptosis of retinal cells that precedes the development of T2DR [37] (Figure 1). Various cytokines (e.g., IL-1β) and growth factors (e.g., VEGF) have also been noted to mediate the proinflammatory role of the PI3K/Akt and ERK1/2 signaling pathway and the angiogenesis in the retina of diabetic animals [38, 39]. Extracellular high-mobility group box-1 (HMGB-1) functions also as a pro-inflammatory cytokine and exhibits angiogenic effects [40]. In the retina of a diabetic, HMGB-1 possibly interacts with advanced glycation end products (RAGE) and activates ERK1/2 and NF-κB to generate an inflammatory response and disrupt the retinal vascular barrier [41, 42]. Preventing the progression of T2DR *via* inhibiting ROS/ angiogenic factors/MAPK might afford the ability to regulate the activation of MMP-2 and -9. Recently, several studies have shown that the clinical expression of T2DR is not only focused on hyperglycemia but also related to obesity, dyslipidemia, blood pressure, and body mass index (BMI), all characteristics for metabolic syndromes. That is, oxidative stress may be due to hyperglycemia in diabetes patients and hyperlipidemia simultaneously. The improved oxidation of fatty acids leads to enhanced NADH/ NAD^+^ ratio and results in the activation of a mechanism like hyperglycemia; it also induces ROS and several signaling pathways to prevent neovascularization and fibrosis in the retina [43, 44].

## 3. Candidate Genes/Pathways

### 3.1. Protein kinase cAMP-dependent (PKA) pathway

The cyclic nucleotide adenosine monophosphate (cAMP) and the PKA have been shown to be intimately involved in hormone action in the metabolic pathways of mammalian cells as well as the regulation of various cellular functions in many cell types [45]. cAMP is particularly important for processes of glucosestimulated insulin secretion among the intracellular signals (phospholipid- derived molecules, the influx and mobilization of Ca^2+^, *etc*.) [46]. cAMP action on insulin secretion is mediated through PKA (serine/threonine kinase) phosphorylation. The regulation of cAMP signaling, including PKA and the exchange protein activated by cAMP2 (Epac) are also important in regulating the secretion of Glucagon-like peptide-1 (GLP-1, stimulates insulin secretion from pancreatic β cells after eating). GLP-1 improves β-cell mass, promotes glucose disposal in streptozotocin-induced diabetic mice, and defends against cardiac microvascular injury of diabetes *via* a cAMP/PKA/Rho-dependent mechanism [47, 48] (Figure 1). Other direct targets of cAMP that are associated with cell proliferation are the MAPK and ERK cascade that provide important crosstalk between hormones and growth factor signaling [49]. An important downstream effector of PKA is the transcription factor cAMP response element binding (CREB). This leads to the recruitment of the CREB binding protein (CBP/p300) and the active expression of Wnt target genes.

### 3.2. G protein and G protein receptor signal

Activation of G protein-coupled receptors’ (GPCRs) ability to regulate multiple signaling pathways include guanine nucleotide exchange factors (GEFs) for Ras and Rho GTPases, MAPKs, PI3K/AKT/mTOR, and their numerous downstream cytosolic and nuclear targets (Figure 1). GPCRs are also the second messenger that triggers the activation of the heterotrimeric G protein (composed of α, β, and γ subunits) to bind GTP and replaces GDP to activate or inhibit a variety of proteins and enzymes. This signaling network contributes to normal cell growth, survival, differentiation, and migration. But, unusual activation of GPCRs/G proteins and their downstream targets can lead to tumor initiation, progression, and metastasis [50]. Changes to GPCRs function may cause many congenital and acquired diseases such as retinitis pigmentosa (rhodopsin mutations), nephrogenic diabetes insipidus (vasopressin receptor mutations), and obesity (melanocortin receptor mutations) [51]. Recent research has demonstrated that the G protein-coupled receptor 91 (GPR91) of retinal ganglion neurons induces the release of VEGF in an oxygen-induced retinopathy rat model and modulates the high glucose-induced VEGF release of RGC-5 cells by inhibiting ERK1/2 and JNK MAPK signaling [52]. Some evidence showed that peptide-binding G-protein-coupled receptors (peptide-binding GPCRs) play an important role in the pathophysiology of vascular dysfunction of diabetes and that this could possibly be a therapeutic target in the treatment of diabetic vasculopathy [53].

### 3.3. Wnt-catenin signaling

Human Wnts regulate a complex signaling cascade in various cell types during both development and disease [54]. The crucial role of Wnts is their participation in a diverse number of fundamental cellular processes (such as cell fate determination during embryonic development, cell proliferation, cell cycle arrest, differentiation, apoptosis and tissue homeostasis) in development and organismal homeostasis. So far, Wnt pathways are commonly known as: (1) the canonical Wnt pathway, which acts through the transcriptional activity of β-catenin; (2) the non-canonical Wnt pathway (planar cell polarity pathway), which involves the activation of Rho small GTPases and JNK and ROCK kinases, and the calcium pathway with the activation of PKC, calmodulin kinase, calcineurin, and the nuclear factor of activated T-cells (NFAT) transcription factor to affect cytoskeleton and cell shape [55]; and (3) the non-canonical Wnt/calcium pathway [56]. The Wnt pathway contains a network of extracellular and intracellular molecules. In canonical Wnt signaling, Wnt glycoproteins bind to membrane heterodimeric receptors (Frizzled and LRP5/6) and recruit the scaffold protein (Dishevelled), then disrupt the β-catenin destruction complex (GSK-3β, apc, and Axin) [57] (Figure 1). Finally, stabilized β-catenin accumulates in the cytoplasm and subsequently translocates to the nucleus to the active complex and drives the expression of Wnt target genes (VEGF, connective tissue growth factor, ICAM-1, and TNF-α). Past studies have shown that secreted frizzled-related protein 4’s (SFRP4, the putative Wnt-binding site of Frizzled proteins) release form is stimulated by interleukin-1β and once stimulated causes reduced glucose tolerance and suppresses insulin exocytosis in T2D patients [58]. Wnt pathway activation has been demonstrated to be associated with nitrosative stress and peroxynitrite formation in DR in humans and in animal models. Current knowledge suggests that the blockade of Wnt pathway might result in pathogenic effects in DR [59].

### 3.4. Androgen receptor nuclear signaling

Androgens are male sex hormones that regulate male development and physiological processes, particularly in the maintenance of the male reproductive system [60]. The biological effects of androgens interact with AR to induce the transcription of a variety of target genes and then regulate downstream androgen-dependent signaling pathways. Histone-modifying enzymes compose one major group of AR coregulators including acetylation, methylation, phosphorylation, ubiquitination, glycosylation, and ADP ribosylation. The main biologically active form of endogenous androgens is testosterone through the action of a P450 family member (commonly known as 5α-reductase). Androgen is an important factor in body composition at age-associated changes in *men*—for instance, in an increase in fat mass and a decrease in lean body mass [61]. Epidemiological studies have shown a bidirectional relationship between low testosterone levels and obesity in men [62]. Yu *et al*. suggests that androgen-deprivation therapy could manage metabolic complications associated with prostate cancer *via* tissue-selective modulation of AR signaling and treatment with insulin-sensitizing agents [63].

### 3.5. Clathrin-coated vesicle cycle review

Clathrin-coated vesicles (CCVs) are named after the protein that is encapsulated into a cage formed by the interaction of Clathrin molecules [64]. The endocytosis is characterized by the internalization of molecules from the cell surface into intracellular membrane compartments [65]. The Clathrin molecule is called a triskelion because it has the ability to concentrate coated vesicles, and this produces a varied protein and lipid load in the nascent vesicle. The major component of isolated coated vesicles is adaptor protein (AP) complexes (AP2). AP2 ligands linked to Clathrin molecule have been identified to mediate endocytosis that a part of synaptic vesicle recycling in the brain extracts [66, 67]. Most material internalized into eukaryotic cells follows the pathway of clathrin-mediated endocytosis, which includes essential nutrients, [iron and cholesterol (bound to transferrin and lowdensity lipoprotein, respectively)], signaling molecules (epidermal growth factor and transforming growth factor beta), and immune complexes, *etc*. [68]. CCVs forming at the plasma membrane are responsible for clathrin-mediated endocytosis (CME) to select protein and lipid cargo for endocytic entrance into the cells. CME also controls the plasma membrane levels of G-protein and tyrosine kinase receptors and is required to couple these receptors to specific intracellular signaling pathways (proliferation, differentiation, cell survival, migration) [69]. CCVs form at the trans- Golgi network. They are protein transport from the secretory pathway to the endosomal and lysosomal system.

### 3.6. γ-Aminobutyric acid (GABA)

The physiological importance of GABA is as an inhibitory neurotransmitter in the central nervous system. Thus far, at least three types of GABA have been characterized as either ionotropic (GABA_A_ or GABA_C_) or metabotropic (GABA_B_) receptors. Ionotropic GABA receptors mediate fast GABA response and belong to ligand-gated chloride channels. Metabotropic GABA_B_ receptors are in the C family of G-protein-coupled receptors (GPCRs) and mediate slow GABA responses which regulate G-proteins and their downstream signaling [70] (Figure 1). Simultaneously, GABA_B_ receptors have been found in the mammalian central nervous system and have become key therapeutic targets for certain neurological diseases. GABA_B_ receptors are composed of GABA_B1_ and GABA_B2_ isoforms that have distinct ligand-binding capabilities. The extracellular domain (ECD) of GABA_B1_ is able to attach to endogenous neurotransmitter (GABA) and receptor ligands (agonists and antagonists), while the ECD of GABA_B2_ has no ligand-binding activity [71]. The phosphorylation of GABA_B_ receptors regulates inwardly rectifying Ca^2+^ and K^+^ channels as well as the activities of adenylyl cyclase (AC) and phospholipase C (PLC) [72]. GABA_B_ receptors may also activate PI3K and MAPK signaling pathways. GABA_B_ receptors have been reported to participate in cell proliferation, migration, and tumor development. GABA_B_ receptor activation inhibits the proliferation and migration of various human tumor cells and inhibits the expression of CREB (a classic downstream effector of PKA) and ERK in tumor cells [73]. Recently, GABA has been suggested to be one of the substances involved in the regulation of immune cell activity and inflammation by inhibition of major inflammatory events mediated by different immune cells. GABA_A_ agonists have been shown to reduce macrophage cytokine production and to inhibit T-cell proliferation, whilst GABA_B_ agonists have been shown to decrease TNF-α production from peripheral blood mononucleated cells and inhibit the release of IL-6 and IL-12 from microglial cells [74, 75]. Oral GABA treatment has ameliorated the inflammatory process both in non-obese diabetic mice and in a mouse model of rheumatoid arthritis [76, 77].

## 4. Conclusion

DR is related to several metabolic abnormalities which are implicated in its pathogenesis. However, the pathogenetic mechanisms of DR are very complex, and the exact mechanisms remain to be determined. Hence, future research must confirm whether diverse candidate genes or pathways are implicated in the pathogenesis of T2DR. It seems need a discovery of novel genetic markers-based therapeutic strategies with the possibility for the prevention and treatment of diabetic vascular complications.
